# Exploring Tobacco Use in Young Childhood Cancer Survivors: the Role of Social Acceptance, Workplace Environments, and Stress Reduction

**DOI:** 10.1007/s10900-025-01493-3

**Published:** 2025-07-18

**Authors:** Anamara Ritt-Olson, Julia Stal, Franceskrista Morales, Parsa Khawari, Lisa Leiby, Tracy Tran, Hien Phuong Le, Joel E. Milam

**Affiliations:** 1 Department of Health, Society, & Behavior, Joe C. Wen School of Public Health, UC Irvine, California, USA; 2 Dana-Farber Cancer Institute, Harvard University, Cambridge, USA; 3 Department of Epidemiology, Joe C. Wen School of Public Health, UC Irvine, California, USA

**Keywords:** Childhood cancer survivors, Tobacco use, Social integration, Workplace norms, Stress coping, Latine health, Qualitative research

## Abstract

Recent advancements in childhood cancer treatment have significantly improved survival rates, yet survivors continue to face considerable morbidity and mortality, rendering them a vulnerable population. Tobacco use, linked to heightened cancer risk, is discouraged among survivors for long-term health preservation; however, many persist in its use, mirroring rates among their non-cancer peers. Through 25 in-depth interviews, we explored the interplay of perceived risk and social integration on tobacco use post-treatment among both Latine and non-Latine young adult survivors. Sixty percent of respondents were male, 44% identified as Latine, with ages ranging from 22 to 38 years (mean age 30 years; mean age at diagnosis 9 years), representing diverse cancer types. Despite awareness of tobacco-related health hazards, including general and respiratory health impacts, addiction potential, and financial burdens, survivors consistently cited stress alleviation as a primary motive for tobacco use. Fearof cancer recurrence was seldom cited as a deterrent, rather survivors identified tobacco as a means to foster peer connections, particularly in workplace settings, where smoking norms prevailed. Co-workers influenced initiation, and smoking breaks served as crucial socializing opportunities, endorsed as acceptable coping mechanisms. Notably, generational factors overshadowed cultural influences on tobacco use. These insights underscore the need for targeted interventions, in which clinicians can emphasize alternative stress management strategies over risk-centric messaging, prioritize social skill development, and address workplace environments as high-risk settings.

This project was supported by TRDRP grant number 28IR-0052 from the National Institute on Minority Health and Health Disparities, P30CA014089. This work was additionally supported by the CERES network funded by the Jacobs Foundation. The content is solely the responsibility of the authors and does not necessarily represent the official views of the National Institutes of Health. Data availability: Due to the nature of this research, participants of this study did not agree for their data to be shared publicly, so supporting data is only available through detailed request to the principal investigator. The authors report no conflict of interest.

Recent advancements in childhood cancer treatment have significantly improved survival rates, yet survivors continue to face considerable morbidity and mortality, rendering them a vulnerable population. Tobacco use, linked to heightened cancer risk, is discouraged among survivors for long-term health preservation; however, many persist in its use, mirroring rates among their non-cancer peers. Through 25 in-depth interviews, we explored the interplay of perceived risk and social integration on tobacco use post-treatment among both Latine and non-Latine young adult survivors. 60% of respondents were male, 44% identified as Latine, with ages ranging from 22 to 38 years (mean age 30 years; mean age at diagnosis 9 years), representing diverse cancer types. Despite awareness of tobacco-related health hazards, including general and respiratory health impacts, addiction potential, and financial burdens, survivors consistently cited stress alleviation as a primary motive for tobacco use. Fear of cancer recurrence was seldom cited as a deterrent, rather survivors identified tobacco as a means to foster peer connections, particularly in workplace settings, where smoking norms prevailed. Co-workers influenced initiation, and smoking breaks served as crucial socializing opportunities, endorsed as acceptable coping mechanisms. Notably, generational factors overshadowed cultural influences on tobacco use. These insights underscore the need for targeted interventions, in which clinicians can emphasize alternative stress management strategies over risk-centric messaging, prioritize social skill development, and address workplace environments as high-risk settings.

## Background

Continuing treatment advances for childhood and adolescent cancer have resulted in more than 80% of patients achieving long-term survival [1]. However, cancer treatments often lead to other serious issues, including developing chronic health problems and experiencing early mortality [2]. These health problems, referred to as “late effects,” are defined as any adverse medical or psychosocial outcome that develops or persists after treatment [2]. By 25 years following treatment, over two-thirds of childhood cancer survivors (CCS) have developed at least one clinically significant late effect, and over one-third have developed one or more late effects classified as severe or life-threatening [2]. Further, CSS experience an eight to eleven-fold risk of premature death compared to the general population [3–5]. Most CCS today remain at increased life-long risk for health problems associated with lower quality of life and premature death [6].

Adolescent and young adult childhood cancer survivors (AYA CCS) face elevated risks of premature morbidity and mortality, with poor health behaviors—particularly substance use—further compounding these risks [6]. Clinical guidelines underscore the importance of promoting health-protective behaviors in this population, including the avoidance of tobacco and other substances, to mitigate the long-term effects of cancer and its treatment [7]. Despite this guidance, an estimated 16–29% of AYA CCS report tobacco use, highlighting a critical gap in survivorship care. Recent work by Kim et al. (2024) contributes important nuance to this literature, revealing racial and ethnic differences in substance use patterns among survivors [8]. Their study found that Latine CCS were more likely to report e-cigarette use than non-Hispanic peers—an alarming trend given the growing prevalence of vaping among youth and its emerging health risks. This finding aligns with broader research indicating that CCS may engage in different patterns of substance use than their peers without a cancer history [9], with implications for both clinical screening and tailored prevention. Tobacco use, in particular, is associated with poorer overall health, an increased risk of developing secondary chronic conditions, and lower quality of life among survivors [10]. Taken together, these data underscore the urgent need for culturally responsive, developmentally tailored, and long-term behavioral health interventions to address substance use within this vulnerable and heterogeneous population.

### Perceived Risk

Despite being aware of the general health risks associated with tobacco use, such as impacts on overall health, respiratory issues, addiction potential, and financial costs, cancer survivors may not consistently connect these risks to an increased personal risk of cancer recurrence. Individuals generally have some awareness of the health risks associated with tobacco use, including an increased risk of cancer, respiratory issues, and addiction, however the perception of these risks often depends on factors such as education, age, and personal health experiences [11]. There can be a disconnect between the acknowledgment of general health risks and the perception of personal risk, suggesting even when individuals are aware of the adverse effects of tobacco on health, they may not always associate these risks with their own well-being including CSS’s not understanding the true extent of health risks associated with cancer treatment [12]. This particularly noted among younger survivors [13].

### Emotion and Smoking

Studies report tobacco use as a coping mechanism to manage stress, which may influence their risk perception, as stress relief may take precedence over concerns about long-term health consequences [14]. There is ample evidence that adolescents and young adults see tobacco use to regulate emotional states, to manage stress, or to alleviate depressed mood [15]. Our recent work revealed a connection between tobacco use and depressive symptoms among survivors, indicating that mental health and substance use are intricately connected [8, 16]. National surveys report that having depression and severe psychological distress were strongly associated with increased tobacco use among adults in the United States [17]. Siqueira and colleagues (2000) found an association between perceived stress and smoking in non-cancer experiencing adolescent Latine which was more pronounced among monthly smokers than experimenters. Therefore, findings suggest cigarette use as a means of stress relief is a prominent form of self-medication [18].

### Peers and Tobacco Use

Prevention efforts rely on a thorough understanding of the predictors associated with the early onset of tobacco use. One of the most prominent factors of adolescent smoking is peer influence. Research across ethnic groups [19] and genders [20] finds that a “pro tobacco” peer environment is highly predictive of smoking. The uptake of tobacco is a social process, and initiation commonly occurs in same sex groups [21]. Thus, prevention efforts commonly attempt to change social norms of tobacco use to reduce future use.

### Current Work

We conducted a series of structured interviews among cancer survivors who used and did not use tobacco in any form with the aim to contextualize the role that perceived risk, mental health, and desire to integrate with peers plays in the decision to engage in substance use post-treatment among culturally diverse young adult survivors. This work will hopefully help delineate theories of tobacco use which in turn will shape interventions and policies to prevent tobacco use among this vulnerable population.

## Methods

### Measures

Our respondents were drawn from the Project Forward Cohort [22] and were selected for participation based on substance use, identified gender, and ethnicity. We sought a wide representation of cancer types and intensity. We further report here on a select few findings. Our measures include demographic information such as current age, gender identity, birthdate, age at diagnosis, race/ethnicity, and treatment intensity. The ethnicity indicator was limited to Latino/Hispanic as a response option. As this was the item endorsed, we refer to our respondents in the results section as that descriptor. To measure the intensity of prior cancer treatment, we used the Intensity of Treatment Rating Scale 3.0 (ITR-3) [23], a validated scale derived from cancer registry data and medical chart review. Treatment is categorized by four levels of intensity: 1 = least intensive (e.g., surgery only), 2 = moderately intensive (e.g., chemotherapy or radiation), 3 = very intensive (e.g., two or more treatment modalities), and 4 = most intensive (e.g., regimens for relapsed disease including hematopoietic cell transplantation). We also measured their connection with healthcare providers. We asked about having a primary care provider and being connected with specialized follow-up care for cancer survivors.

We also asked about their history of tobacco or electronic cigarette use. To measure depressive symptoms, we used the well-validated screener for depression, the Center for Epidemiologic Studies Depression Scale (CES-D) [24]. We employed the cut-off of 16 for indication of being at risk for true depression.

### Research Team

Six total interviewers conducted the interviews. All interviewers attended three hours of initial training and weekly meetings to review interviews. Additionally, interviewers completed ethics training and certifications were obtained in both general ethical research and cancer specific research. Interviewers sent confirmation emails after each interview reviewing the interview and highlighting any issues that occurred. One interviewer was a PhD faculty member white and female. Two were PhD students both female, one white non-Latine and one Asian. Three interviewers were advanced MPH graduate students, two female and non-Latine white and one was Latine male. One Asian Female interviewer was a dual MPH and Masters in Social Work student. Participants and researchers had no contact with each other prior to the interview. All interviewers had personal interest in the subject with various personal connections to cancer experiences from significant others, friends, to being cancer survivors themselves. Several interviewers also had strong personal interests in substance abuse and use.

### Sampling

Purposive sampling grouped participants according to preselected criteria relevant to a particular research question. Respondents were chosen based on their surveyed responses of past substance use. We selected these from participants in the Project Forward Cohort, a population-based study of 1237 CCS. Participants were selected across ethnicity, cancer type, and age. Sixty participants were selected from our cohort study of young adult survivors of childhood and adolescent survivors– Project Forward (recruitment and participants explained in Milam, et al.). We scheduled 27 interviews but completed 25 interviews. Two participants were scheduled and lost to follow-up. We recruited 25 total participants (44% Latine ethnicity; 36% female) emerging/young adult survivors of childhood and adolescent cancers aged 22–38 years old with a representative blend of cancer types (e.g., acute lymphoblastic leukemia, lymphoma) who are stages 2 + and well enough to participate.

### Interviews

A PhD student with knowledge of the database selected possible cases at random that fit our inclusion criteria and have complete contact information. Cohort participants have already consented to be contacted by us for additional research efforts. Possible cases were given in small batches of 5 to our callers who set up appointments for the 20–60 min interviews.

Semi-structured interviews were employed with additional probes. Our interview guide is described in Table 1. Qualitative interviews lasted between 20 min and 55 min. Interviews were performed in private locations where the respondent could not be overheard. The interviewer used a digital recorder to record the session and then uploaded that recording to OTTER which transcribes the recording into text suitable for use with coding software. Alternatively, the interviewer called the participant using Zoom conference line that records and transcribes the interview. Upon completion of the interview the interviewer saved the interview recording and transcript into a password-protected file. Zoom Pro is HIPPA compliant and has been since 2017. It is commonly used for telemedicine by medical providers. This link explains HIPPA compliance in detail https://zoom.us/docs/doc/Zoom-hipaa.pdf.

**Table 1:** Interview guide

Question	Follow up questions
First of all how are you today? Can you share with me a bit about your health history?	We want to understand what the cancer experience was like for you it can be different for so many people. You do not need to go into a lot of detail.
How many of your friends use tobacco regularly?	How common is it? How pervasive and accepted? How often do they use?
If yes, when did you first start? Tell me more about that first experience? If no, would you ever? How do you use it? Do you smoke it? Vape it? Chew it? Do you use it with other substances?	Have you ever used any **tobacco** products?
Do you ever feel pressured to use tobacco with them? Or drink more than 3 drinks?	Do you feel left out if you aren’t using?
What are the good things and the bad things that come from tobacco use?	What are some of the reasons people say they use different tobacco products? How much is too much?
Why do people use tobacco? Or drink 3 or more drinks in one sitting?	To cope? To deal with stress? To manage symptoms?
Do you think there are cultural differences in the ways people think about tobacco use?	Does it vary by how long people have been in the country?
Have you or has a friend ever had a problem that could have been from using tobacco?	Like the reports of breathing issues from vaping, bronchitis, lost motivation, lost a job…
How does your family view tobacco use?	What would they say about using?
How would important others in your life view Tobacco use?	Like a romantic partner? Your best friend? Your boss? Your teacher?
What do you think other people of your culture or ethnic group’s general attitude is towards tobacco use?	What do people in general all around think?
Do you think there are cultural differences in the ways people think about tobacco?	Does it vary by how long people have been in the country?

### Analyses

We utilized a grounded theory approach [25] to explore the perceptions of tobacco use. Transcripts were reviewed for accuracy by research staff and volunteers who had not conducted the interviews. They listened to the recordings and updated the written transcripts.

An initial code book was drafted from our interview guide and then four transcripts were reviewed by the study team in order to assemble a refined coding scheme that was directed by the exploration of the specific phenomena of substance use by cancer survivors. The initial list was condensed and organized into codes and series of sub codes. Initially two study staff coded 5 transcripts and compared the coding agreement. They then reviewed and adapted the code book. The codes were further refined and definitions expanded. Staff coded 15 more transcripts, with an inter-rater reliability of 0.84. Another student reviewed the codebook and all the transcripts to further refine the codebook for a final version. All transcripts were cleaned and uploaded into nVivo. A final coding with the final version of the codebook and all transcripts were then completed with that final codebook.

### Sample Characteristics

We interviewed a total of 25 individuals who had experienced cancer, and they experienced a wide variety of cancer types including brain (3), ALL (4), acute myeloid leukemia (2), Hodgkins (3), Melanoma (2), Other central nervous (1), urinary system (1). Sample characteristics are presented in Table 2, wherein sex, race, and ethnicity were requested, with ethnicity being described as Latino/Hispanic or not.

**Table 2:** Sample characteristics

	*N* (%) or M (SD)
**Demographic characteristics**	
Age at interview	30.08 (5.20)
*Sex*	
Male	15 (60.0)
Female	10 (40.0)
*Race*	
White	20 (80.0)
Middle Eastern	2 (8.0)
Asian/Pacific Islander	2 (8.0)
Other	1 (4.0)
*Ethnicity*	
Hispanic/Latino	11 (44.0)
Non-Hispanic/Latino	14 (56.0)
*Clinical Characteristics*	
Age at diagnosis	9.4 (6.24)
Type of cancer	
Brain	3 (12.0)
Acute Lymphocytic Leukemia	8 (32.0)
Acute Myeloid Leukemia	3 (12.0)
Hodgkin’s Lymphoma	5 (20.0)
Melanoma	2 (8.0)
Non-Hodgkin’s Lymphoma	1 (4.0)
Urinary Systems	2 (8.0)
Other central nervous system	1 (4.0)
Substance use characteristics*	
Cigarette	12 (48.0)
Alcohol	11 (44.0)
Marijuana	9 (36.0)
Prescription drugs	1 (4.0)
E-Cigarette	5 (20.0)
*Reported use in the past 30 days	

## Results

We refer to our respondents by their assigned numeric code and provide some descriptions below. Through synthesis and interpretation of the transcribed interviews, themes emerged around expectations of tobacco use, peer use, initiation of tobacco use, cultural views of tobacco, and familial perceptions of use. What was said and not said reflected perceptions of tobacco among our respondents. Although the general health risks of tobacco use were acknowledged little connection between personal risk and cancer was mentioned.

### Expectations and Tobacco Use

We asked our participants for examples of both good and bad things that can come from using tobacco. Prompts included asking for reasons people use tobacco. Some participants focused on the health consequences around tobacco use, especially lung functioning. One respondent described being out of breath and having a reduced ability to function physically. A theme emerged that the expectation that tobacco use would negatively impact strength and vitality. One respondent shared that the effects would be detrimental to their physical strength.

A respondent who identifies as male, endorsed being Hispanic/Latino, and was 25 at time of interview, reported never using tobacco but reported having tried marijuana.

Bad things, um, well lung cancer, respiratory issues. Uh, you lose conditioning really, really fast.

Another male identified respondent, endorsed being Hispanic/Latino shared that they saw no benefits from using tobacco and shared that a family member was negatively impacted by tobacco use.

Um, I’ll be honest, I don’t know that I see any good from tobacco use. Uh, the only person in my life that used to smoke is my aunt, and I never saw anything come good of it. It definitely like brought down her health, like she’s had a lot of issues as a result of that. Um, yeah that’s it. That’s really been my only interaction with it.

This respondent had never used any substances, tobacco or marijuana, perhaps a function of being an adolescent when they were diagnosed. They were 17 when they were diagnosed with cancer and underwent more intense treatments, their treatment intensity was a 3 out 4. At the time of interview, they were 38 years old. This respondent was also in follow-up care at the time that they completed the interview. We have seen that being in follow-up care is associated with less habitual tobacco use [8].

After reviewing the transcripts and memos, one striking omission became apparent: very few participants mentioned expecting any personal consequences from tobacco use, particularly in terms of increasing their own cancer risk. For most, the perceived risks of tobacco felt distant and impersonal, with no connection to potential secondary cancer risk. This was especially surprising given that the interviews began by asking respondents to reflect on their cancer journey and overall health. Yet, despite this personal orientation, none of them mentioned avoiding tobacco due to concerns about triggering another cancer diagnosis.

This pattern suggests a disconnect between their own health and the recognized risks of tobacco use. It’s possible this disconnect reflects a shift in priorities, where finding a sense of community takes precedence over health concerns. After surviving cancer during a crucial stage of psychosocial development—often marked by disruptions like missing school, work, and social connections—there seems to be a strong drive to rebuild a social identity. For some, tobacco use may serve as a means of connecting with peers, suggesting that the need for community might outweigh concerns about personal health risks. Additionally, this disconnect may also relate to tobacco being used as a coping mechanism for stress, where the immediate relief it provides can overshadow awareness of long-term risks. If the brain perceives a behavior as helpful for reducing stress, that can often override concerns about potential harm.

### Stress and Tobacco Use

The stress relief offered by tobacco may override the risks summarized earlier. If stress is high and unmanaged, tobacco offers a way to cope. Many respondents, when asked why someone would start to smoke, answered - to relieve stress. The driving force behind tobacco use was to deal with the worry of stress and people felt that it would be an effective means to feel better. This respondent reported that using tobacco was the best feeling ever.

I was probably 18, 19, and I was drunk, and I smoked a cigarette, it was like the best feeling ever. [laughs] But, you know, there you go.

This person identifies as male, endorsed being Latino/Hispanic, and was 33 at time of interview. They reported cigarette use, but not marijuana or electronic use. Our respondents almost universally endorsed tobacco as stress relieving, whether they currently used tobacco or not. Respondents were asked why they think anyone would use tobacco. Again, and again respondents said to relieve stress.

Another male identified, Hispanic/Latino, and was 23 at time of interview cancer reported cigarette use, marijuana use, and electronic tobacco use. When asked why people use tobacco they said simply: “For stress or anxiety. They use it for - just a habit that they have to do.”

A respondent commented that smoking is rumored to help with “I’ve heard people say it helps with stress or it helps curb their appetite”. This respondent is Non- Hispanic/Latino female identified and were 32 at the time of the interview. Another female identified Non-Latino/Hispanic who was 38 at time of interview, has smoked cigarettes, used marijuana, and but not electronic cigarettes also described tobacco as “ Probably just a stress reliever”.

Respondent said that stress leads one down a dangerous path.

I definitely think it’s a way for people to deal with stress. I think tobacco is incredibly addictive, um, like I think weed can be addictive but I don’t know that it’s like the weed itself or like a lifestyle tied to, to it more than like tobacco is a 1000% addictive product and I think some people are more susceptible to addiction. Um, or like addict behavior and things and tobacco is the top one on that list. So, um I think it’s like… it’s a stress reliever but I think it’s also just like an oral fixation….

They are female identified, Non-Hispanic/Latino and were 27 when they completed the interview. They have tried cigarettes and marijuana, but never tried an electronic cigarette.

### Tobacco Initiation and the Power of the Situation

Our respondents shared that they started smoking later than expected in early adulthood. Previously adolescence was seen as a high-risk period for smoking initiation. This could be due in part to the cancer experience; cancer survivors may have had adolescence disrupted by treatments or other restrictions or could be a result of societal shifts. Some interviewees described smoking at work. Smoke breaks were an opportunity to bond with their work peers. Loneliness and a desire to connect seemed to drive smoking at work. Young adults who have experienced cancer have disrupted developmental histories. While others were developing a refined sense of self and building relationships, they were battling illness. They may feel less able to initiate contact due to delayed development of social skills. One of our female identified survivors described above, reported that they started smoking to spend time with their work friends. It started as just a way to feel part of the group and evolved into regular use.

I was 20 uh, probably 20 years old. And I was working in a restaurant and the only reason you get a break when you work in a restaurant is to go outside and smoke a cigarette, so it was like a very cultural thing. I never actually did take the breaks but everyone that I worked with would smoke cigarettes so when we would go out to the bar after work and drink, I would like get a couple puffs of their cigarette, and it’s just like, felt like um I don’t know, like “these are interesting” and then you know, if I, I was never able to smoke one without having any drinks in my system. Um, it just was very unappealing and not nice to me, but it just felt normal. Like that was a time when cigarettes and smoking was still quite popular, um, and I was living in Boston, so a lot of people smoked cigarettes there….

Work culture plays a role in the adoption and regular use of tobacco. With the success of smoking prevention programs in California schools may no longer be seen as a welcome environment for tobacco use. Workplace settings, however, remain tobacco dominant. A male identified, non-Latino/Hispanic who completed our interview at age 26 and described their tobacco initiation.

I was working with this guy. And I don’t know, I just wanted like to have a cigarette. And so, I, he was like yeah, here you go. [laughs] And it was, you know, it’s just kind of an easy way to get a break at work. I think people that have like different career paths, like me, I work construction and there’s plenty of people that you know smoke cigarettes and chew and stuff like that so they, they don’t care. But a lot of other people that work in offices or wherever, you know, they probably don’t like it as much because with us, nobody really cares. It’s already all dirty and nasty, you know.

### Desire to Integrate with Peers

Respondents reported that the desire to form friendships or social connections was a driving force for initial use and themes around tobacco as a conduit to social interaction was reported by smokers.

A male identified and non-Hispanic/Latino who completed our interview at age 32 and described their tobacco use as being independent of their peers.

Umm. I’m probably one of my only, umm, one of the few friends that are around that still smoke tobacco, umm although, still, at a party, with some alcohol, you’ll see even the non-smokers smoking.

Non-users were less likely to report tobacco use by their peers. Few respondents said that they friends smoked or vaped tobacco, but here again work played a strong role in tobacco use. This individual stressed that work and career influences tobacco use.

And people have like different career paths, like me, I work construction. There’s plenty of people that you know smoke cigarettes and chew and stuff like that so they don’t care. A lot of other people that work in offices or wherever. You know, they probably don’t like it as much because with us, nobody really cares it’s already all dirty and nasty, you know.

While a respondent who is male identified and non-Hispanic/Latino and completed our interview at age 37, described their tobacco use being shaped by their experience in the military and incarceration.

When I was 17, I joined the army and a lot of guys in there chew tobacco and so I started chewing tobacco. And then you know I quit smoking at about 17 or 18 and started chewing tobacco. I didn’t use any tobacco while I was in jail. That’s another thing that’s really common in jails tobacco, chewing tobacco. I didn’t use that because that was part of my whole sobriety. I was trying to you know Purge myself of anything like that. When I got home, I started using chewing tobacco again.

### Family Influence

Many studies have previously found that peers had a greater influence on health behaviors than family for adolescents and young adults. Our respondents reported nearly universal disapproval of smoking behavior by their families but did not specify that fear of cancer recurrence was the driving force behind the disapproval. This mirrors the lack of connection between cancer recurrence and tobacco use we noted with the respondents in general. Several respondents referred to parental and family disapproval as powerful. This reposndent shared that their family does disapprove of cigarettes, but they do report using cigarettes themselves.

Cigarettes they think are terrible. Umm. But my father smokes cigars and my mom likes to enjoy a cigar with him, very rarely but she does. Umm other than that, my side of the family just completely non-smokers. My wife’s side of the family, all smokers.

### Culture and Generational Differences

When questioned about the intersection of culture and tobacco use, most respondents failed to identify a clear connection. However, as illustrated by the above respondent, who has military and incarceration experiences, some cultural variations were observed in specific environments. Reflecting on their observations, this individual also stated that:

You know I noticed a lot of you know white guys would chew tobacco or would smoke it. A lot of Black guys would smoke tobacco you know smoke. I don’t recall as being as common with Hispanic culture or with them and their race.

Our sample was purposively balanced with half Hispanic/Latino and half non, but our Hispanic/Latino respondents did not report on cultural differences or influences on tobacco. Persian respondents explained how hookah was common in their culture but instead of cultural expectations, respondents connected generation as a factor in tobacco expectations. This suggested that acculturation is a driving force instead of a cultural norm. The acculturation process, broadly defined as the interchange of cultural attitudes and behaviors that occurs when people from differing cultural backgrounds come into contact with one another, is complex and multidimensional [26]. Acculturation to the United States culture has been implicated as a risk factor for unhealthy behaviors among Latine adolescents. These unhealthy behaviors include alcohol and other drug use [27], as well as smoking [28]. In contrast, our respondents suggested that this pattern has shifted with acculturation as a protective factor. Older generations were seen as more accepting of tobacco use in general. This respondent alluded to this in their experience.

And then for older generations, it’s more just normal. Because where I work, where I work, a lot of older people will smoke, they will take smoke breaks and stuff like that….Yeah. So I think it’s more like an age issue probably also, younger people probably see it more negatively than other people do.

While another shared the importance of generation on tobacco use.

I think for the younger generation is more negative like type of view. For older generations, it’s more just normal. Because where I work, older people will smoke, smoke during breaks and stuff like that and younger people won’t. I think it’s more like an age issue probably also, younger people probably see it more negatively than other people do. The marketing and the times before tobacco was seen as something to just relax make no big issue, and over time and it changed with surgeon general warnings.

Another respondent went on to explain that other countries’ attitudes towards tobacco could be seen in the advertisements and promotion of tobacco products which suggested that tobacco use was normative. They are male identified and Hispanic/Latino.

“Yeah, because if you just got here from another country where cigarette smoking was prevalent and you get here and you see all these new billboards, or a lot of new billboards, meaning that there is no propaganda for it anymore, or other new propaganda is uh, indicating that it causes cancer and stuff like that. So, yeah, it can change your opinion, or they could like, think different or whatever.”−43.

Respondent 422 is male identified and non-Hispanic/Latino. They completed our interview at age 30 and refrain from using all substances. They commented on the differences between countries and generations.

Oh, I mean I think if you’re coming from a culture where has a different role like us okay that’s like an entirely different like medium and a different like cultural thing, you know, and like that can be a significant part in some cultures and if you come recently then they’ll probably have more meaning for you. But if you’re a few generations away from that you probably have more American views of the use and ideas about the benefits of tobacco.

Older generations grew up in a time when tobacco use was more culturally accepted and widespread, often before the full health risks were widely known. As a result, smoking may be more normalized for them due to these early influences. In contrast, attitudes toward tobacco use have shifted significantly over the years, with younger generations being exposed to more comprehensive health education and awareness campaigns. This increased knowledge has led to a generally more negative view of tobacco use among younger people. Understanding these generational shifts is crucial for designing effective public health interventions. By recognizing the distinct influences on each age group’s attitudes toward tobacco, we can better tailor anti-smoking strategies to address their specific beliefs and behaviors. This approach ensures that interventions are more relevant and effective in reducing tobacco use across diverse age groups. Older family members may need education outreach to understand the negative impact of tobacco on cancer survivors.

## Discussion

Cancer survivors are at particular risk for subsequent cancers and should avoid tobacco. We interviewed several individuals from diverse backgrounds who had experienced cancer about their perceptions of tobacco use. The study explored perceptions of tobacco use among respondents, focusing on themes such as expectations, perceived risk, initiation, cultural views, familial influences, and stress relief. A key finding was the lack of a strong connection between tobacco use and personal cancer risk, highlighting a disconnect between general health awareness and the recognition of specific cancer-related risks. Factors like workplace culture perceived benefits of smoking, and acculturation were significant influences within this population, pointing to the need for targeted, long-term behavioral interventions. This is especially notable given their risk for cancer recurrence.

Workplace culture contributed to tobacco initiation, as smoke breaks were viewed as opportunities for social bonding with peers. This perceived benefit of building social connections was a significant factor, with respondents noting that tobacco use often began in emerging adulthood rather than in early adolescence. Rather than simply enforcing a blanket ban on smoking, exploring the underlying reasons for smoking in workplace settings could lead to more effective strategies for reducing tobacco use.

Stress relief also emerged as a key motivator for tobacco use, with respondents describing it as an effective coping mechanism, despite being aware of health risks like shortness of breath. Respondents also highlighted negative effects on physical strength and vitality due to tobacco use but consistently emphasized its stress reduction properties. The lack of a strong connection between tobacco use and personal cancer risk was also emphasized when participants shared personal experiences of family members suffering health issues because of tobacco use, but having little acknowledgement of how tobacco use was risky for them. The fear of cancer recurrence was also not explicitly identified as a reason for discouragement among respondents’ family disapproval.

Cultural and generational differences were noted, with older generations being perceived as more accepting of tobacco use. In particular, Persian respondents mentioned the expected behavior of hookah use, meanwhile Latine respondents did not specifically address cultural influences on tobacco. The study highlighted the impact of generational attitudes and acculturation on tobacco expectations. Our respondents suggested that this pattern has shifted with acculturation as a protective factor. This suggestion requires much more research as it counters decades of findings.

### Implications for Theoretical Frameworks

A grounded theory approach in qualitative research aims to refine, expand, or develop models that explain health behaviors. Many existing theories highlight the role of peer attitudes toward smoking as a critical factor in predicting regular smoking, with peer influence recognized as a key driver of adolescent smoking for decades. Our findings indicate that peer influence extends beyond adolescence into young adulthood and that context matters. Young adults are transiting to new workplace contexts, and this can be both stressful and require strong social skills. These insights suggest a need to deprioritize perceived risk as a primary barrier to tobacco use and instead revisit the role of emotion and mood. Traditional frameworks, such as the Theory of Planned Behavior and the Health Belief Model, often overlook mood regulation, which may be a significant factor in tobacco use.

### Implications for Providers

Clinicians and other care providers may benefit from adopting a more holistic approach that moves beyond risk-centric messaging to address the underlying emotional and social factors contributing to tobacco use. Emphasizing alternative stress management strategies, such as mindfulness, relaxation techniques, or physical activity, can equip individuals with healthier coping mechanisms. Furthermore, prioritizing the development of social skills may empower individuals to resist peer pressure and navigate complex interpersonal dynamics, especially in high-risk environments like the workplace.

In addition to focusing on the individual, clinicians might consider engaging families in discussions about protective health behaviors. This could involve fostering open communication, encouraging family support systems, and educating family members on how to serve as positive role models or accountability partners. By addressing the broader social and environmental context, clinicians can create a more comprehensive support network to reduce tobacco use and promote long-term well-being.

### Implications for Future Research

In summary, the study revealed complex interactions between personal experiences, societal influences, and health perceptions in shaping respondents’ attitudes toward tobacco use. The disconnect between awareness of general health risks and the specific risk of cancer recurrence suggests a need for targeted education and interventions in this population with time-limited prevention efforts may be better served by focusing on stress reduction, mental health, and peer relationships as opposed to increasing risk perceptions of tobacco use. Survivors consistently recognized a myriad of risks associated with tobacco. They subsequently endorsed tobacco as stress-reducing and described ways that tobacco helped with their peer connections. Survivors reported that their workplace facilitated their smoking. This opens several avenues for intervention. Policy shifts in workplace tobacco are already in motion, but additionally, programs that promote social skills and assistance with opportunities for survivors to form friendships that don’t rely on shared risk-taking could be widely beneficial. Respondents discounted to some extent the influence of parents on tobacco use but did consider their disapproval as powerful. Parental sources of influence should not be discounted. Several theorists contend that peer influence is more relevant than parental factors [29], but parenting practices may be related to the types of friendships that are formed and related to mental health.

## References

[1] Howlader, N., Noone, A. M., Krapcho, M., Miller, D., Brest, A., Yu, M., Ruhl, J., Tatalovich, Z., Mariotto, A., Lewis, D. R., Chen, H. S., Feuer, E. J., & Cronin, K. A. (Eds.). (2019). *SEER Cancer Statistics Review, 1975–2016*. National Cancer Institute.

[2] Oeffinger, K. C., Mertens, A. C., Sklar, C. A., et al. (2006). Chronic health conditions in adult survivors of childhood cancer. *The New England Journal of Medicine*, *355*, 1572–1582. 17035650 10.1056/NEJMsa060185

[3] Mertens, A. C., Liu, Q., Neglia, J. P., et al. (2008). Cause-specific late mortality among 5-year survivors of childhood cancer: The childhood cancer survivor study. *Journal of the National Cancer Institute*, *100*(19), 1368–1379. 18812549 10.1093/jnci/djn310 PMC2556702

[4] Reulen, R. C., Winter, D. L., Frobisher, C., et al. (2010). Long-term cause-specific mortality among survivors of childhood cancer. *Journal of the American Medical Association*, *304*(2), 172–179. 20628130 10.1001/jama.2010.923

[5] Ness, K. K., Armstrong, G. T., Kundu, M., et al. (2018). Premature physiologic aging as a paradigm for Understanding increased risk of adverse health across the lifespan of survivors of childhood cancer. *Journal of Clinical Oncology*, *36*(21), 2206–2215. 29874132 10.1200/JCO.2017.76.7467 PMC6553838

[6] Armstrong, G. T., Kawashima, T., Leisenring, W., Stratton, K., Stovall, M., Hudson, M. M., Sklar, C. A., Robison, L. L., & Oeffinger, K. C. (2014). Aging and risk of severe, disabling, life-threatening, and fatal events in the childhood cancer survivor study. *Journal of Clinical Oncology: Official Journal of the American Society of Clinical Oncology*, *32*(12), 1218–1227. 24638000 10.1200/JCO.2013.51.1055 PMC3986385

[7] Mollica, M. A., McWhirter, G., Tonorezos, E., et al. (2024). Developing National cancer survivorship standards to inform quality of care in the united States using a consensus approach. *Journal of Cancer Survivorship*, *18*, 1190–1199. 38739299 10.1007/s11764-024-01602-6 PMC11324674

[8] Kim, Y., Ritt-Olson, A., Tobin, J., et al. (2023). Beyond depression: Correlates of well-being in young adult survivors of childhood cancers. *Journal of Cancer Survivorship*, *17*, 1397–1404. 35187609 10.1007/s11764-022-01186-z PMC11999713

[9] Ruiz, M. E., Sender, L., Torno, L., & Fortier, M. A. (2016). The associations of age and ethnicity on substance use behaviors of adolescent and young adult childhood cancer survivors. *Psycho-Oncology*, *25*(10), 1229–1236. 27434382 10.1002/pon.4225

[10] Kaul, S., Veeranki, S. P., Rodriguez, A. M., & Kuo, Y. F. (2016). Cigarette smoking, comorbidity, and general health among survivors of adolescent and young adult cancer. *Cancer*, *122*(18), 2895–2905. 27286172 10.1002/cncr.30086

[11] Gritz, E. R., Talluri, R., Fokom Domgue, J., Tami-Maury, I., & Shete, S. (2020). Smoking behaviors in survivors of Smoking-Related and Non–Smoking-Related cancers. *JAMA Netw Open*, *3*(7), e209072. 10.1001/jamanetworkopen.2020.9072 32614423 10.1001/jamanetworkopen.2020.9072 PMC7333020

[12] Kahalley, L. S., Robinson, L. A., Tyc, V. L., Hudson, M. M., Leisenring, W., Stratton, K., Mertens, A. C., Zeltzer, L., Robison, L. L., & Hinds, P. S. (2012). Risk factors for smoking among adolescent survivors of childhood cancer: A report from the childhood Cancer survivor study. *Pediatric Blood & cancer*, *58*(3), 428–434. 10.1002/pbc.23139 21618409 10.1002/pbc.23139 PMC3165077

[13] Ford, J. S., Puleo, E., Sprunck-Harrild, K., et al. (2014). Perceptions of risk among childhood and young adult cancer survivors who smoke. *Supportive Care in Cancer*, *22*, 2207–2217. 24659242 10.1007/s00520-014-2165-8 PMC10360447

[14] Bradford, N. K., McDonald, F. E. J., Bibby, H., Kok, C., & Patterson, P. (2022). Psychological, functional and social outcomes in adolescent and young adult cancer survivors over time: A systematic review of longitudinal studies. *Psycho-Oncology*, *31*(9), 1448–1458. 10.1002/pon.5987 35734846 10.1002/pon.5987 PMC9544373

[15] González-Yubero, S., Lázaro-Visa, S., & Palomera Martín, R. (2020). The protective association of trait and ability emotional intelligence with adolescent tobacco use. *International Journal of Environmental Research and Public Health*, *17*(18), 6865. 32962216 10.3390/ijerph17186865 PMC7558078

[16] Stal, J., Ramirez, C. N., Huh, J., et al. (2023). Examining the factor structure of an adapted posttraumatic growth inventory in a sample of childhood cancer survivors. *Evaluation & the Health Professions*, *46*(1), 100–104. 10.1177/01632787221109309 35727145 10.1177/01632787221109309 PMC11974463

[17] Cornelius, M. E., Loretan, C. G., Jamal, A., et al. (2023). Tobacco product use among Adults– United states, 2021. *Mmwr. Morbidity and Mortality Weekly Report*, *72*, 475–483. 10.15585/mmwr.mm7218a1 37141154 10.15585/mmwr.mm7218a1 PMC10168602

[18] Siqueira, L., Diab, M., Bodian, C., & Rolnitzky, L. (2000). Adolescents becoming smokers: The roles of stress and coping methods. *Journal of Adolescent Health*, *27*(6), 399–408. 10.1016/s1054-139x(00)00167-1 11090742

[19] Landrine, H., Richardson, J. L., Klonoff, E. A., et al. (1994). Cultural diversity in the predictors of adolescent cigarette smoking: The relative influence of peers. *Journal of Behavioral Medicine*, *17*, 331–346. 7932684 10.1007/BF01857956

[20] Harrell, W. (1998). Gender and equity issues affecting educational computer use. *Equity & Excellence in Education*, *31*(3), 46–53.

[21] Friedman, L. S., Lichtenstein, E., & Biglan, A. (1985). Smoking onset among teens: An empirical analysis of initial situations. *Addictive Behaviors*, *10*(1), 1–13. 4003131 10.1016/0306-4603(85)90048-6

[22] Milam, J., Slaughter, R., Tobin, J. L., et al. (2018). Childhood cancer survivorship and substance use behaviors: A matched case-control study among Hispanic adolescents and young adults. *Journal of Adolescent Health*, *63*, 115–117. 10.1016/j.jadohealth.2018.02.005 PMC6070351 30060847

[23] Werba, B. E., Hobbie, W., Kazak, A. E., Ittenbach, R. F., Reilly, A. F., & Meadows, A. T. (2007). Classifying the intensity of pediatric cancer treatment protocols: The intensity of treatment rating scale 2.0 (ITR-2). *Pediatric Blood & cancer*, *48*(7), 673–677. 17427232 10.1002/pbc.21184

[24] Radloff, L. S. (1977). The CES-D scale: A self-report depression scale for research in the general population. *Applied Psychological Measurement*, *1*(3), 385–401.

[25] Charmaz, K., & Belgrave, L. (2012). Qualitative interviewing and grounded theory analysis. In J. F. Gubrium, J. A. Holstein, A. B. Marvasti, & K. D. Mckinney (Eds.), *The SAGE handbook of interview research: The complexity of the craft* (2nd ed., pp. 347–366). SAGE Publications, Inc.

[26] Berry, J. W. (1980). Acculturation as varieties of adaptation. In A. Padilla (Ed.), *Acculturation: Theory, models and findings* (pp. 9–25). Westview.

[27] Epstein, J. A., Botvin, G. J., & Diaz, T. (2001). Alcohol use among Dominican and Puerto Rican adolescents residing in new York city: Role of Hispanic group and gender. *Journal of Developmental & Behavioral Pediatrics*, *22*(2), 113–118. 11332780 10.1097/00004703-200104000-00005

[28] Unger, J. B., Cruz, T. B., Rohrbach, L. A., et al. (2000). English Language use as a risk factor for smoking initiation among Hispanic and Asian American adolescents. *Health Psychology*, *19*(5), 403–410. 11007148 10.1037//0278-6133.19.5.403

[29] Harris, J. R. (1995). Where is the child’s environment? A group socialization theory of development. *Psychological Review*, *102*(3), 458–489.

